# Supplementary material for: Identification of a Small Molecule Compound Active against Antibiotic-Tolerant Staphylococcus aureus by Boosting ATP Synthesis
Source: Int J Mol Sci. 2023 Mar 26;24(7):6242. doi: 10.3390/ijms24076242 (PMC10094146; doi:10.3390/ijms24076242)
Supplement: Supplementary file 1 [file ijms-24-06242-s001.zip › ijms-2284976-supplementary.pdf]

International Journal of *Molecular Sciences*

SUPPLEMENTARY INFORMATION

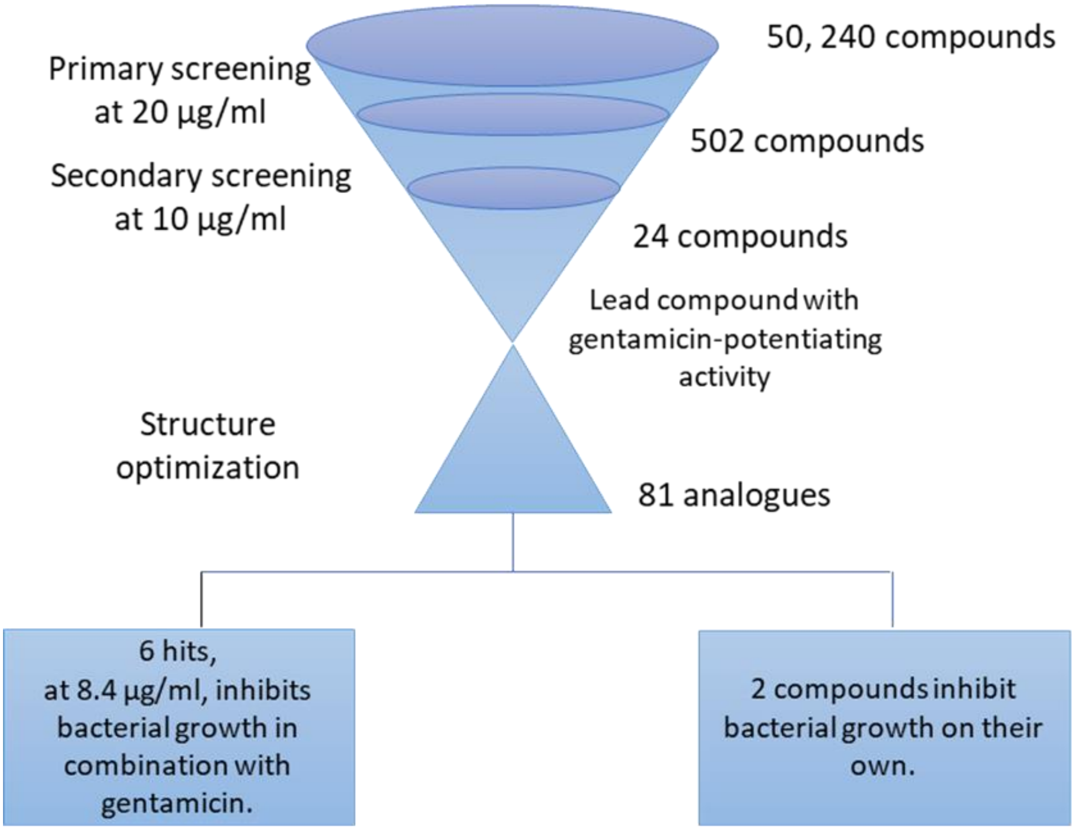

Figure S1. Identification of SA-558 from high-throughput screening. Schematic illustration of processes involved in compound screening of gentamicin-adjuvant.

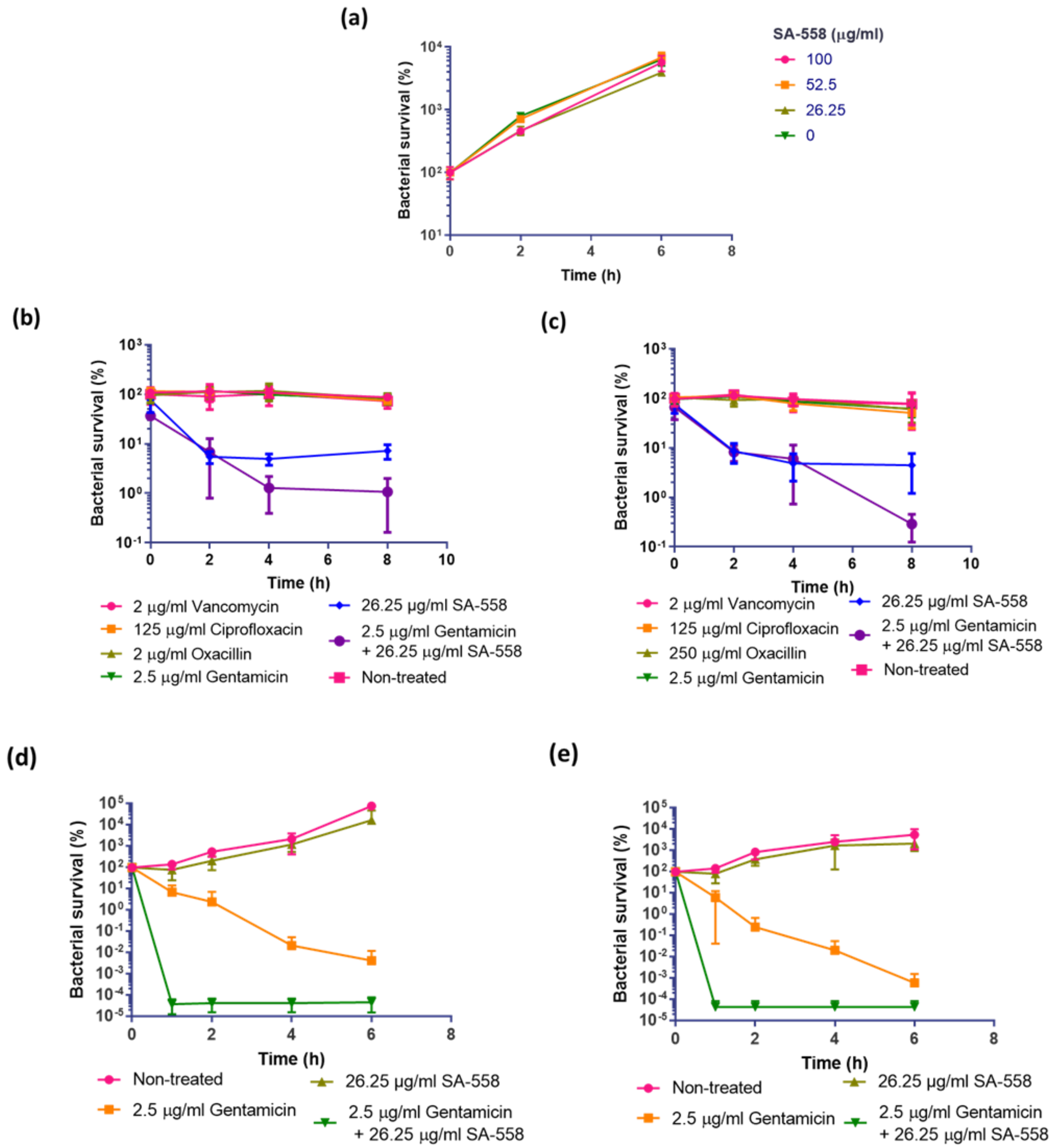

Figure S2. Antibacterial activity of SA-558 and/or gentamicin against *S. aureus*. (a) Effect of SA-558, in the range of 26.25 to 100 µg/ml, on exponential phase *S. aureus* Mu3. (b-c) Antimicrobial activity of SA-558 against stationary phase *S. aureus*, (b) USA300-3 and (c) LAC strain. (d-e) Antimicrobial activity of SA-558 against exponential phase *S. aureus*, (d) USA300-3 and (e) LAC strain.

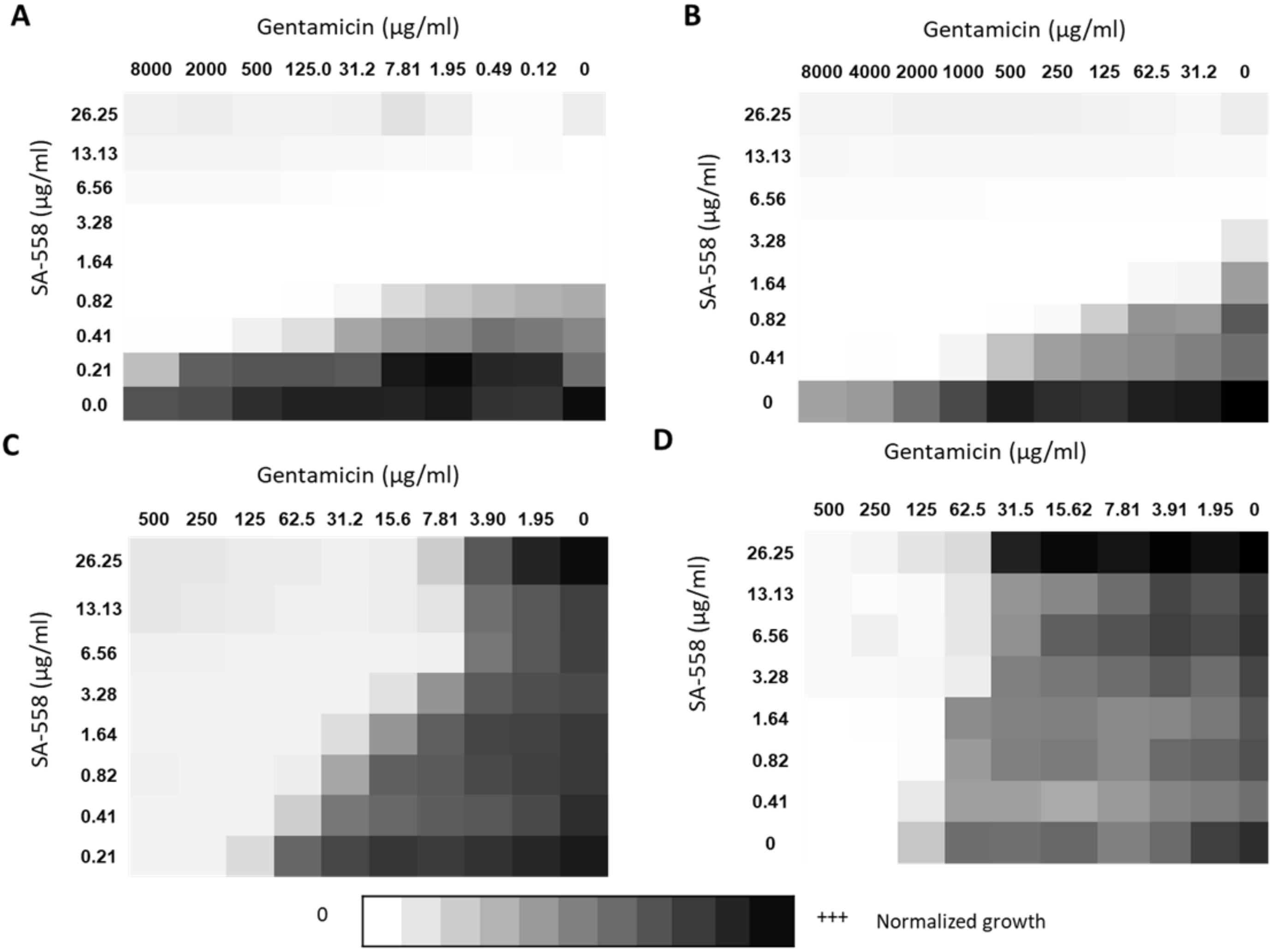

Figure S3. SA-558 gentamicin-potentiating activity in a function of media pH. Checkerboard assays were conducted with media at different pH. (A) 5.5, (B) 6.5, (C) 7.5 and (D) 8.5.

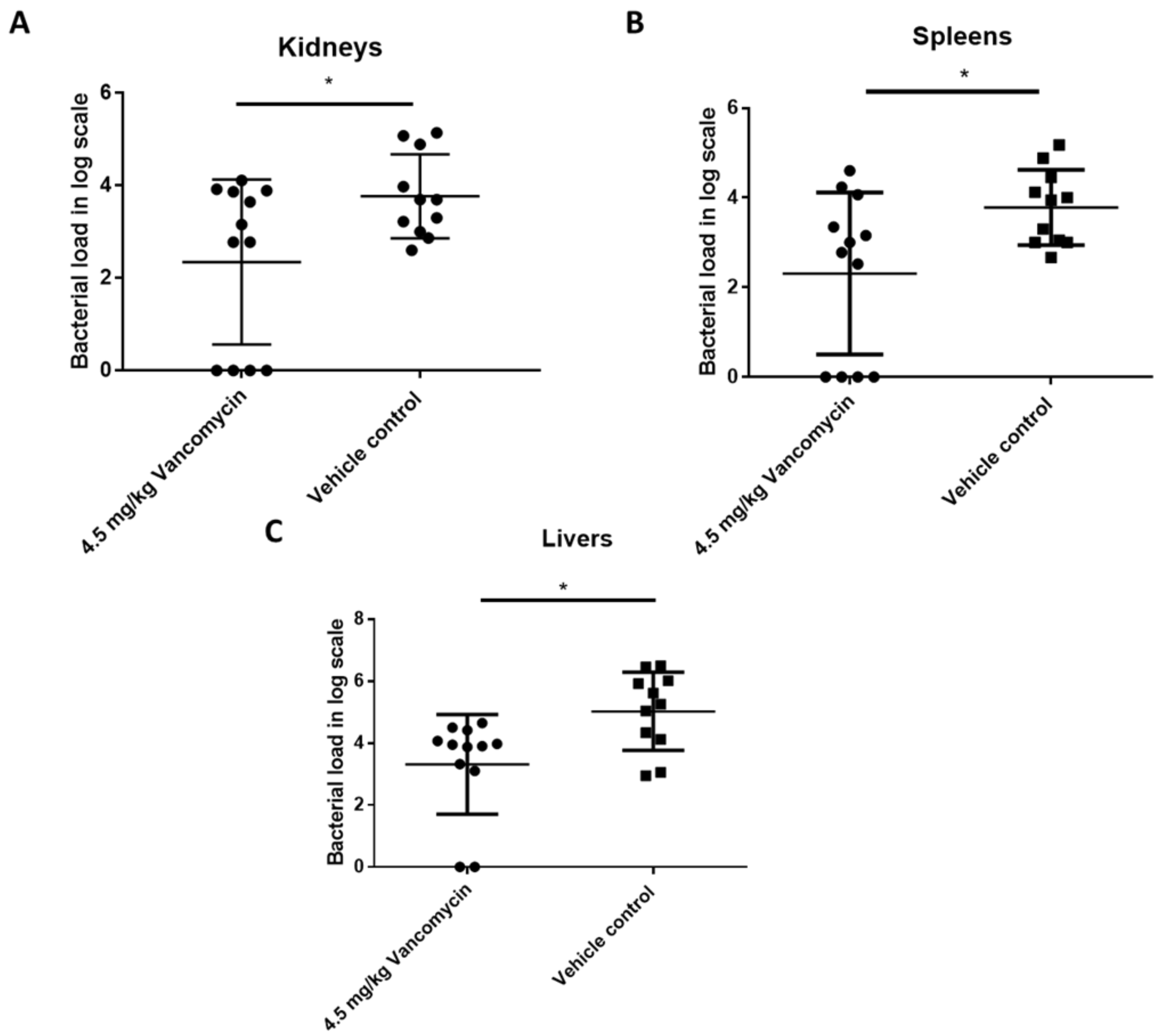

Figure S4. Efficacy of vancomycin, at 4.5 mg/kg against *S. aureus* ST239 IIIAH infection. Comparison of bacterial load in (A) kidneys, (B) spleens, and (C) livers of infected mice (n= 11-12) receiving either 4.5 mg/kg vancomycin and vehicle control. Statistical significance was determined by Student's t test (* $p < 0.05$).

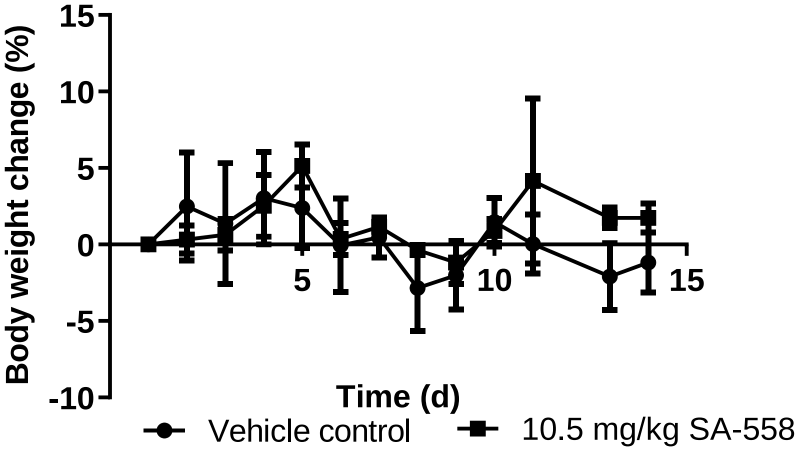

Figure S5. Body weight change of mice (n =3) of 10 doses of SA-558 treatment without infection.

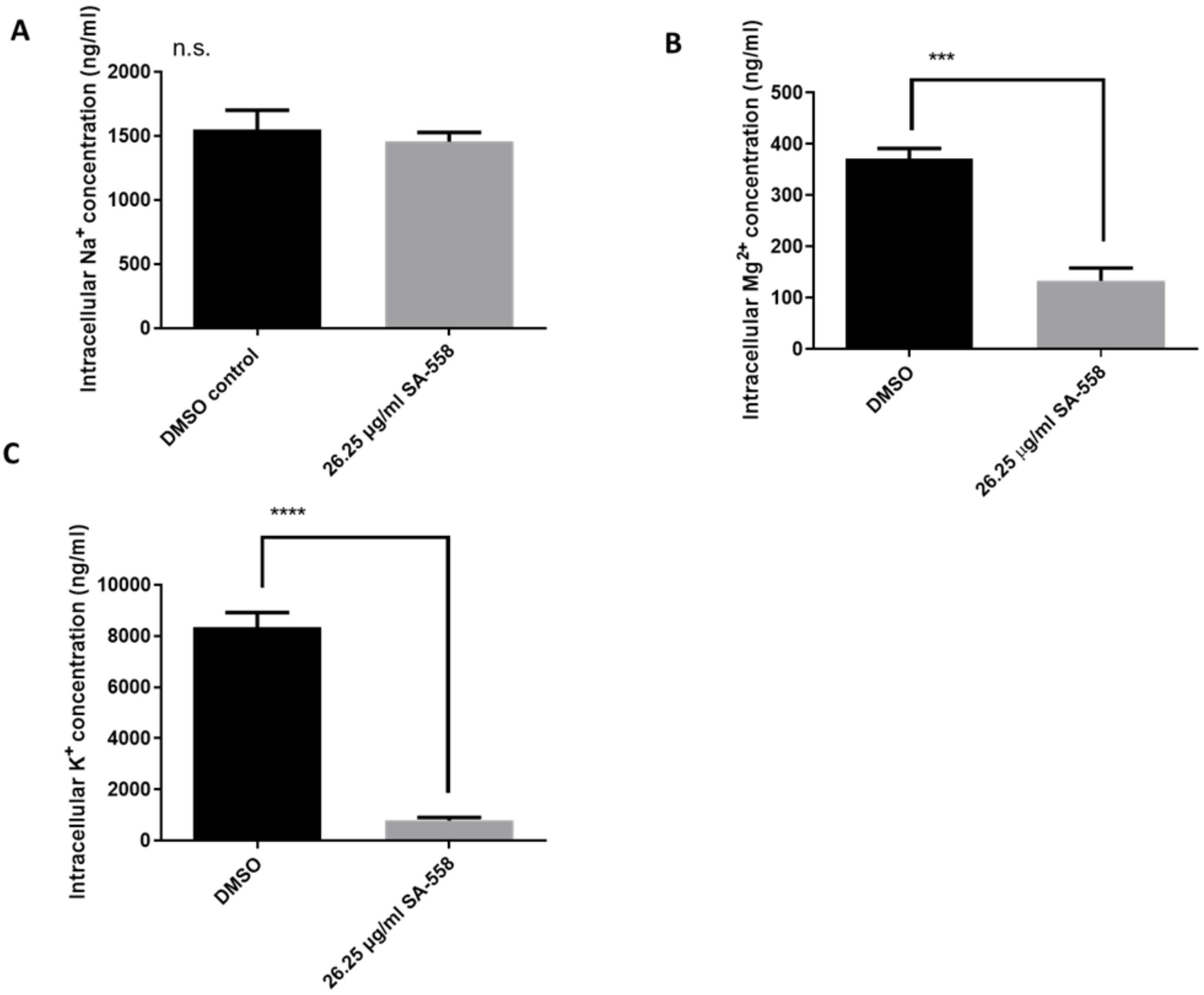

Figure S6. SA-558 effect on intracellular metal ion concentration in *S. aureus* Mu3, (A) Na+, (B) Mg2+, and (C) K+. Statistical significance was determined by Student's t test (*** $p < 0.001$ and ****$p < 0.0001$).

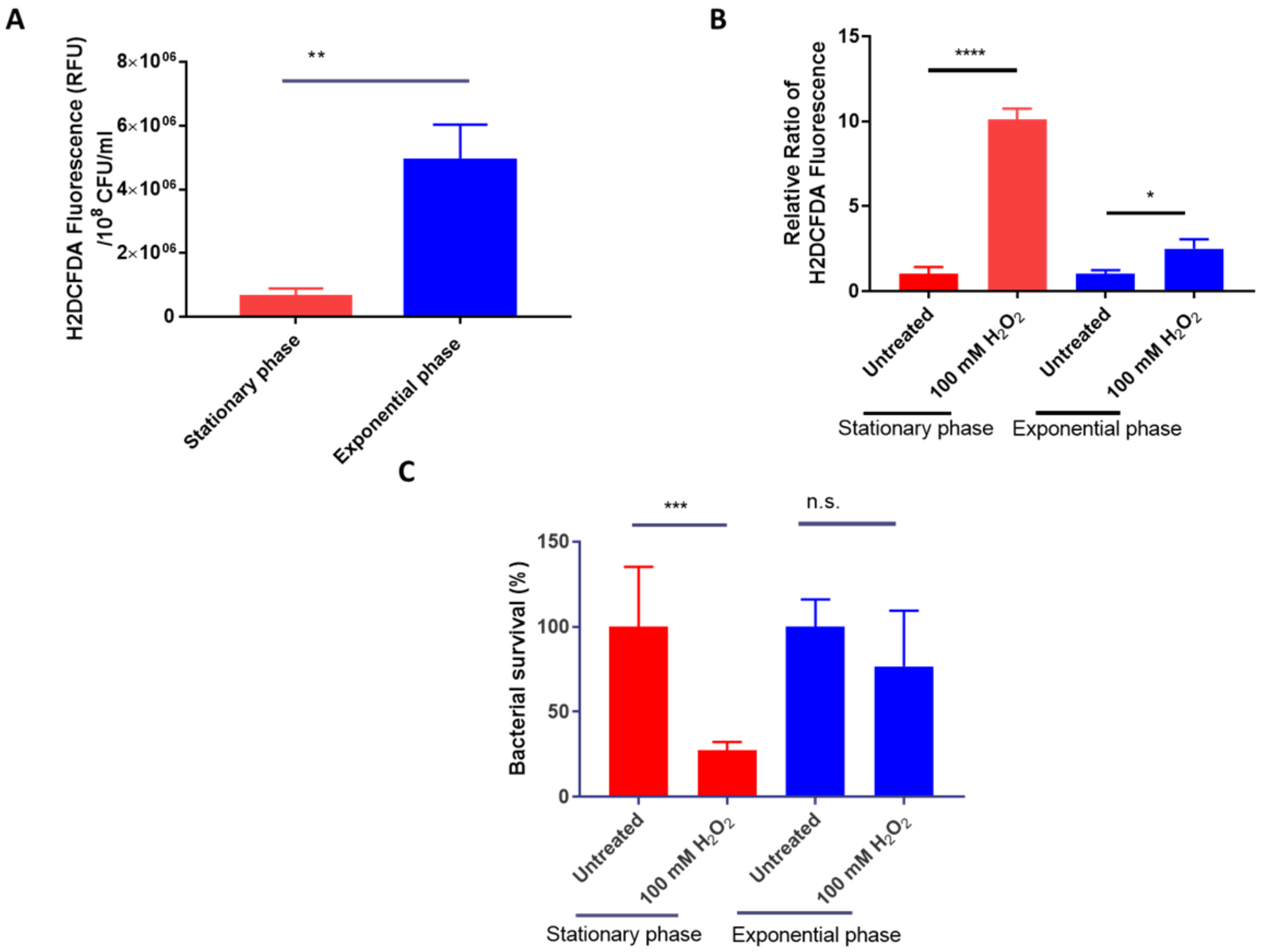

Figure S7. Comparison of antioxidant capacity of stationary phase and exponential phase *S. aureus*. (A) Intracellular ROS level in stationary phase and exponential phase *S. aureus* Mu3. (B) The effect of H_2O_2 on ROS induction in stationary phase and exponential phase *S. aureus* Mu3. ROS dye, H2DCFDA was used for ROS measurement. (C) The effect of H_2O_2 on bacterial survival of *S. aureus* Mu3 in stationary phase and exponential phase *S. aureus*. Statistical significance was determined by Student's t test (* $p< 0.05$, ** $p< 0.01$ and ***$p< 0.001$, ****$p< 0.0001$). Bars represent the mean ± SD.

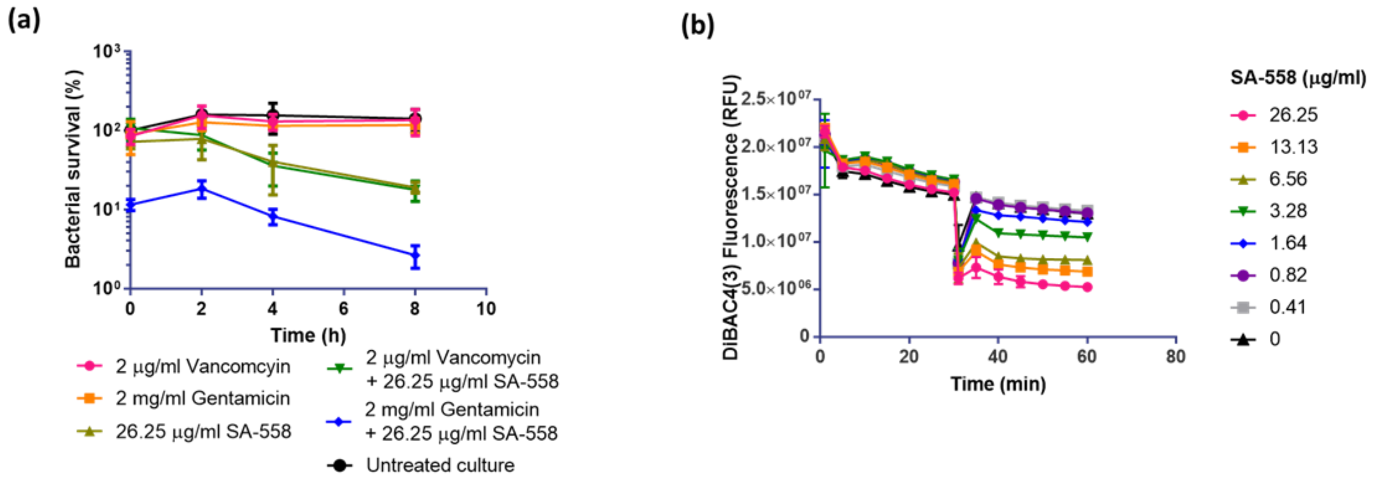

Figure S8. SA-558 activity on the *S. aureus* ST239 IIIAH strain. (a) Antimicrobial activity of SA-558 against the stationary phase culture; (b) Effect of SA-558 on membrane potential.

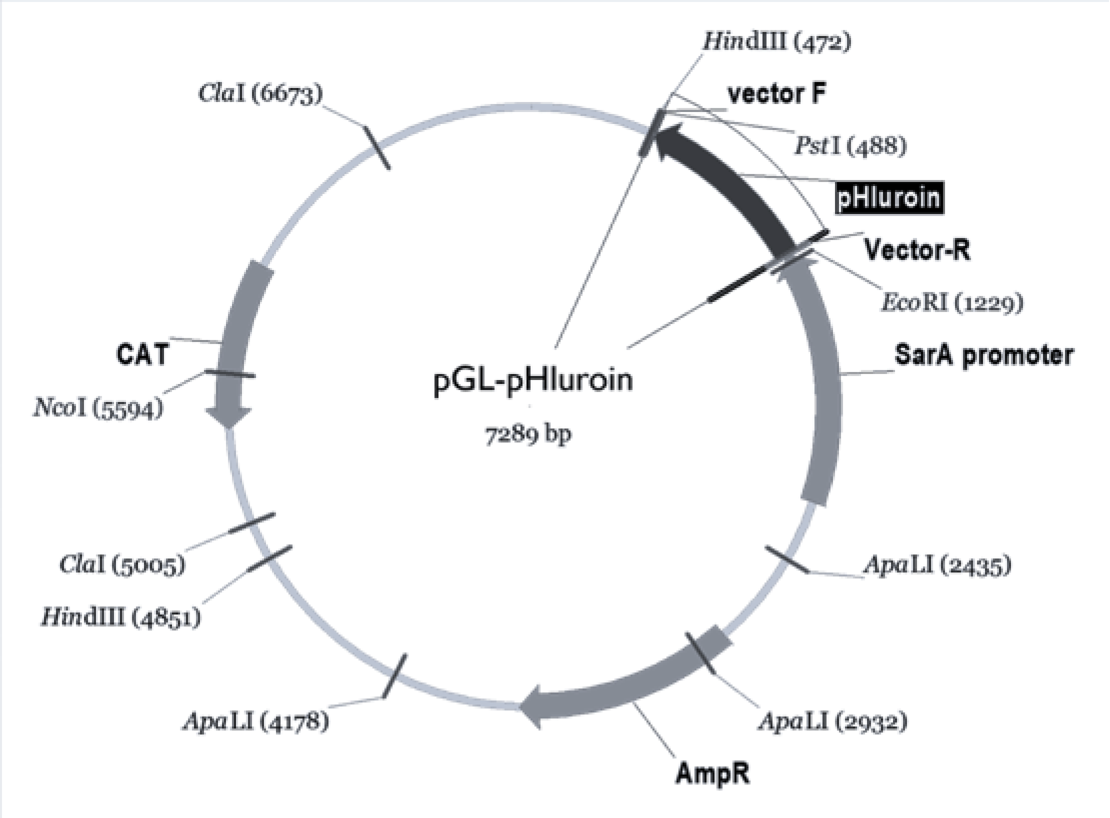

Figure S9. Plasmid map of pGL-pHluorin for intracellular pH measurement. pHluorin was amplified from pUC57, and the vector was amplified from pGL-sarA, resulting in pGL-pHluorin.

Table S1. Antibacterial activity of compounds at 8.4 μg/ml with or without gentamicin in *S. aureus* Mu3.

Cpd no.	BHI only	With 100 μg/ml Gentamicin
1	Bacteria grown	Bacteria grown
2	Bacteria grown	Bacteria grown
3	Bacteria grown	Bacteria grown
4	Bacteria grown	Bacteria grown
5	Bacteria grown	Bacteria grown
6	Bacteria grown	Bacteria grown
7	Bacteria grown	Bacteria grown
8	Bacteria grown	Bacteria grown
9	Bacteria grown	Bacteria grown
10	Bacteria grown	Bacteria grown
11	Bacteria grown	Bacteria grown
12	Bacteria grown	Bacteria grown
13	Bacteria grown	No growth
14	Bacteria grown	Bacteria grown
15	Bacteria grown	Bacteria grown
16	Bacteria grown	Bacteria grown
17	Bacteria grown	No growth
18	Bacteria grown	Bacteria grown
19	Bacteria grown	No growth
20	Bacteria grown	No growth
21	Bacteria grown	Bacteria grown
22	Bacteria grown	Bacteria grown
23	Bacteria grown	Bacteria grown
24	Bacteria grown	Bacteria grown
25	Bacteria grown	Bacteria grown
26	Bacteria grown	Bacteria grown
27	Bacteria grown	Bacteria grown
28	Bacteria grown	Bacteria grown
29	Bacteria grown	Bacteria grown
30	Bacteria grown	Bacteria grown
31	Bacteria grown	Bacteria grown
32	Bacteria grown	Bacteria grown
33	Bacteria grown	Bacteria grown
34	Bacteria grown	Bacteria grown
35	Bacteria grown	Bacteria grown
36	Bacteria grown	No growth
37	Bacteria grown	Bacteria grown
38	Bacteria grown	Bacteria grown
39	Bacteria grown	Bacteria grown
40	Bacteria grown	Bacteria grown
41	Bacteria grown	Bacteria grown
42	Bacteria grown	Bacteria grown
43	Bacteria grown	Bacteria grown
44	Bacteria grown	Bacteria grown
45	Bacteria grown	Bacteria grown
46	Bacteria grown	Bacteria grown
47	Bacteria grown	Bacteria grown
48	Bacteria grown	Bacteria grown
49	Bacteria grown	Bacteria grown
50	Bacteria grown	Bacteria grown
51	Bacteria grown	Bacteria grown
52	Bacteria grown	Bacteria grown
53	Bacteria grown	Bacteria grown
54	Bacteria grown	Bacteria grown
55	Bacteria grown	Bacteria grown
56	No growth	No growth

57	Bacteria grown	Bacteria grown
58	Bacteria grown	Bacteria grown
59	Bacteria grown	Bacteria grown
60	Bacteria grown	Bacteria grown
61	Bacteria grown	Bacteria grown
62	Bacteria grown	Bacteria grown
63	Bacteria grown	Bacteria grown
64	Bacteria grown	Bacteria grown
65	Bacteria grown	Bacteria grown
66	Bacteria grown	Bacteria grown
67	Bacteria grown	Bacteria grown
68	Bacteria grown	Bacteria grown
69	Bacteria grown	Bacteria grown
70	Bacteria grown	Bacteria grown
71	Bacteria grown	Bacteria grown
72	Bacteria grown	Bacteria grown
73	Bacteria grown	Bacteria grown
74	Bacteria grown	Bacteria grown
75	Bacteria grown	Bacteria grown
76	Bacteria grown	Bacteria grown
77	Bacteria grown	Bacteria grown
78	Bacteria grown	Bacteria grown
79	Bacteria grown	Bacteria grown
80	No growth	No growth
81	Bacteria grown	Bacteria grown
SA-558	Bacteria grown	No growth
DMSO	Bacteria grown	Bacteria grown

Table S2. Comparison of the gentamicin-potentiating activity and TC_{50} of the first-tier hits. [a] Fold of reduction in gentamicin MIC in the presence of 26.25 μg/ml SA-558. Gentamicin MIC was determined in *S. aureus* Mu3 strain. [b] TC_{50} was determined in Vero cell line by using MTT assay.

Compound(s)	Potentiation (fold) [a]	TC_{50} (μg/ml)[b]
SA-558	32	>105
13	32	29.4
17	16	17.6
19	16	39.5
20	8	5.0
36	32	28.6

Table S3. Antibiotic Susceptibility of *S. aureus* strains used in this study.

Antibiotic(s) / *S. aureus* strain(s)	MIC (μg/ml)				
	**Vancomycin**	**Ciprofloxacin**	**Oxacillin**	**Gentamicin**	**SA-558**
Mu3	<2	31.25	1000	250	>26.25
ST239 IIIAH	<2	62.5	1000	500	>26.25
USA300-3	<2	31.25	1	0.625	>26.25
LAC	<2	31.25	250	0.625	>26.25

Table S4. Bacterial strains used in this study.

Species/ Strain	RKC no.	Description	Source/ Reference
S. aureus			
Mu3	329	hVISA clinical isolate from Juntendo University Hospital (JUH) in 1996.	Lab collection/[1]
ST239 IIIAH	504	MRSA clinical isolate, Sequence Type 239, SCCmec type IIIAH.	Lab collection
ATCC 29213		Wichita, reference strain isolated from human.	Lab collection
USA300-3		CA-MRSA strain	Lab collection/[2]
USA300 LAC		CA-MRSA strain	Lab collection/[3]

Table S5. Plasmid used in this study.

Plasmid	Relevant characteristics	Source/ Reference
pGL	Plasmid with dual lux and GFP reporter for gene expression using pACL2084 backbone.	[4]
pGL-SarA	SarA promoter was cloned into pGL for gene expression control and used as DNA template for vector preparation in pGL-pHluorin cloning.	[4]
pUC57	pHluorin was codon optimized for *S. aureus* and cloned into plasmid.	Synthesized by Genescript.
pGL-pHluorin	Codon-optimized pHluorin was cloned into vector prepared from pGL-SarA for constitutive expression under control by SarA promoter.	This study

Table S6. Primers used in this study.

RKP no.	Sequences (5'-3' direction)	Purpose and Remarks
pH-R	CTTGCATGCCTGCAGTTATTTATATAATTCATCC ATACCATGTGTAATACCTGCTGC	PCR amplification of pHluorin (with pH-F) from pUC57.
pH-F	ACTAGGGAGGTTTTAAACATGTCAAAAGGTG AAGAATTATTTACAGGTGTTGTTC	PCR amplification of pHluorin (with pH-R) from pUC57.
2122	CTGCAGGCATGCAAG	PCR amplification of vector from pGL-sarA (with RKP 2123)
2123	GTTTAAAACCTCCCTAGTCGACG	PCR amplification of vector from pGL-sarA (with RKP 2122)
2124	CGTTATACAAATTTTAACCCTGTTAGG	Clone checking primer for pGL-pHluorin (with RKP 482)
482	CAGCACATCCCCCTTTC	Clone checking primer for pGL (reverse).

SUPPLEMENTARY METHODS

High-throughput screening

Two sets of plates were set up for compound screening, one with BHI medium only and one with a sub-MIC of gentamicin. Compounds were assayed at a final concentration of 20 μg/ml in 384-well microtiter plates (Greiner Bio-One) in primary screening. *S. aureus* Mu3, at 5 x 10^5 cfu/ml per well, was added and incubated at 37 ℃ for 16 hours. Bacterial growth was monitored by OD_{595} using the DTX 880 multimode detector. Compounds with antibacterial activity in combination with gentamicin were selected as hits. In subsequent screening, compounds were assayed at lower concentrations with the same experimental procedures by primary screening to identify more potent hits.

Checkerboard assay

Checkerboard assays were used to evaluate the interaction between identified hits and antibiotics [5] in conditions according to the CLSI guidelines [6]. Briefly, 2-fold serial dilutions of the selected compound were combined with 2-fold serial dilutions of the tested antibiotic, making an 8 x 10 matrix in a 96-well microtiter plate. The interaction between the compounds tested and antibiotic was visualised by heat map, in which bacterial growth increases with color intensity. To vary the media pH, hydrochloric acid and sodium hydroxide were added for titration.

Cytotoxicity test

Cytotoxicity of the compounds was evaluated with an MTT assay as previously described [7]. Briefly, Green monkey kidney cells, Vero, were seeded at a density of 4x10^4 cells/well in Eagle's minimal essential medium (MEM) supplemented with 10 % Fetal Bovine Serum (FBS) 1 day prior to the assay. Two-fold dilutions of compounds, including dimethyl sulfoxide (DMSO) control, were prepared in plain MEM and added to the cells. After a 24-hour incubation at 37 ℃, 5% CO_2, MTT solution, at 5 mg/ml, was added and incubated for an additional 4 hours, followed by cell lysis with lysis buffer (10% SDS, 0.01 M HCl). On the next day, OD_{570} with reference to OD_{640} was determined in a DTX 880 Multimode Detector (Beckman Coulter, Brea, CA, USA). MTT reading relative to control was calculated for toxic concentration determination.

In vivo testing in acute peritonitis murine model

The *S. aureus* peritonitis murine model was established with reference as previously described [8]. The bacterial inoculum was prepared by sub-culturing the overnight culture of multidrug resistant *S. aureus* ST 239 IIIAH to the BHI medium at a ratio of 1:100, shaking at 37 ℃, 250 rpm for 2 hours. The culture was harvested at 10, 000 xg for 10 min at 4℃, washed twice with equal volume of PBS, and finally resuspended in 25 ml PBS. CFU was enumerated by plating dilutions of bacterial suspension onto BHI agar plates. Bacterial suspension (1 x 10^8 cfu) was delivered to mice by i.p. injection. At 1 h post-infection, the mice were randomized into different cages and received different antibiotic treatments, including vancomycin, at 4.5 mg/kg, and the vehicle control. The drug treatments were administered twice a day by i.p. injection for 3 days. On the third day, the mice were sacrificed, and organs, including liver, spleen, and kidney, were harvested and homogenized for CFU enumeration. The experiment was repeated twice, and results were pooled for statistical analysis.

Intracellular metal ion concentration

An overnight culture of *S. aureus* Mu3 was sub-cultured in BHI medium and grown for 2 hours at 37°C, with shaking. The culture was then exposed to treatment of SA-558 or DMSO as a control for 30 min incubation and analyzed as described previously [9]. Briefly, the bacterial cells were then harvested, washed twice with MilliQ water, and finally

resuspended in MilliQ water. The suspension was dried overnight at 60 °C using a centrifuge evaporator. Dried cells were solubilized in 200 µl 69% HNO3 and lysed by incubation at 95 °C for 1.5 hours. A quantity of 1% HNO_3 was added and resuspended by sonication. The suspension was then transferred to 1.5 ml tubes using a 1 ml syringe and filter by 0.22 µm PTFE filter. Samples were prepared for inductively coupled plasma mass spectrometry (ICP-MS) by diluting 20-fold with 1% HNO_3. Na+, Mg2+, and K+ concentrations were calculated from the standard curve.

ROS measurement

To compare antioxidant capacity of stationary phase and exponential phase *S. aureus*, the response of the culture towards H_2O_2 was characterized. In previous studies, H_2O_2 was shown to induce ROS [10] and activate defense mechanism in bacteria [11]. The bacterial culture was adjusted to 10^8 cfu/ml and loaded with 5 µM H2DCFDA. The overnight culture was harvested, washed, and resuspended in M9 minimal medium, and the suspension was used as stationary phase bacteria. The overnight culture was sub-cultured at 1:100 in LB medium and grown for 2 hours, and the suspension was used as exponential phase bacteria. The culture was then exposed to 100 mM H_2O_2 for 30 min. An aliquot of 200 µl was added to the 96-well black opaque plate (SPL) for fluorescence measurement by using a SpectraMax Paradigm Multi-Mode Microplate Reader (Molecular Devices, San Jose, CA, USA). An aliquot of 100 µl was taken for serial-dilution with PBS and spot-plating onto BHI agar for CFU enumeration to evaluate H_2O_2 killing.

REFERENCES

1. Hiramatsu, K., et al., *Dissemination in Japanese hospitals of strains of Staphylococcus aureus heterogeneously resistant to vancomycin.* Lancet, 1997. **350**: p. 1670-1673.
2. García-Fernández, E., Koch, G., Wagner, R.M., Fekete, A., Stengel, S.T., Schneider, J., Mielich-Süss, B., Geibel, S., Markert, S.M., Stigloher, C. and Lopez, D., *Membrane Microdomain Disassembly Inhibits MRSA Antibiotic Resistance.* Cell, 2017. **171**(6): p. 1354-1367.
3. Grosser, M.R., Paluscio, E., Thurlow, L.R., Dillon, M.M., Cooper, V.S., Kawula, T.H., and Richardson, A.R., *Genetic requirements for Staphylococcus aureus nitric oxide resistance and virulence. .* PLOS Pathogen, 2018. **14**(3).
4. Gao, P., Wang, Y., Villanueva, I., Ho, P. L., Davies, J., & Kao, R. Y. T. , *Construction of a Multiplex Promoter Reporter Platform to Monitor Staphylococcus aureus Virulence Gene Expression and the Identification of Usnic Acid as a Potent Suppressor of psm Gene Expression.* Frontiers in Microbiology, 2016. **7**.
5. Lorian, *Antimicrobial Combinations, in Antibiotics In Laboratory Medicine*, L.W.a. Wilkins, Editor. 2005: Philadelphia. p. 365-441.
6. CLSI., *Performance Standards for Antimicrobial Susceptibility Testing.* Clinical and Laboratory Standards Institute, 2021. **m100**.
7. Lai, K.K., Cheung, N. N., Yang, F., Dai, J., Liu, L., Chen, Z., … Kao, R. Y., *Identification of Novel Fusion Inhibitors of Influenza A Virus by Chemical Genetics.* Journal of virology, 2015. **90**(5): p. 2690-2701.
8. Gao, P., Davies, J., & Kao, R. , *Dehydrosqualene Desaturase as a Novel Target for Anti-Virulence Therapy against Staphylococcus aureus.* mBio, 2017. **8**(5): p. e01224-17.
9. Martin, J.E., and Giedroc, D.P., *Functional determinants of metal ion transport and selectivity in paralogous cation diffusion facilitator transporters czcd and mnte in streptococcus pneumoniae.* Journal of Bacteriology, 2016. **198**(7): p. 1066-1076.
10. Kohanski, M.A., Dwyer, D.J,, Hayete, B., Lawrence, C.A., Collins, J.J. , *A common mechanism of cellular death induced by bactericidal antibiotics. .* Cell, 2007. **130**(5): p. 797-810.
11. Sen, A., and Imlay, J.A., *How microbes defend themselves from incoming hydrogen peroxide.* Frontiers in Immunology, 2021. **12**.
